# Large Doses of Chloral

**Published:** 1873-07

**Authors:** 


### LARGE DOSES OF CHLORAL.

In the *Medical Times*, March 22, 1873, Dr. Maxwell reports an instance in which a patient accidentally took two hundred and sixty grains of chloral at one dose. He simply slept deeply for twenty-four hours. A case was reported in the same journal, October 15, 1870, in which the patient took four hundred and sixty grains of chloral without fatal effects.—*Detroit Review*, May, '73.

